# Research Progress on the GP3 Protein of Porcine Reproductive and Respiratory Syndrome Virus

**DOI:** 10.3390/ani15030430

**Published:** 2025-02-04

**Authors:** Chen Lv, Zhiyu Yang, Xiaolin Lan, Fang Liang, Weili Kong, Ruining Wang, Mengmeng Zhao

**Affiliations:** 1 Guangdong Provincial Key Laboratory of Animal Molecular Design and Precise Breeding, School of Animal Science and Technology, Foshan University, Foshan 528225, China; lc0214fosu@163.com (C.L.); 20220420212@stu.fosu.edu.cn (Z.Y.); 13538003365@163.com (X.L.); 15726370117@163.com (F.L.); 2 Gladstone Institutes of Virology and Immunology, University of California, San Francisco, CA 94158, USA; weili.kong@gladstone.ucsf.edu; 3 College of Veterinary Medicine, Henan University of Animal Husbandry and Economy, Zhengzhou 450046, China; 80882@hnuahe.edu.cn

**Keywords:** PRRSV GP3, genetic evolution, glycosylation, protein interactions, advances in vaccine research

## Abstract

Porcine reproductive and respiratory syndrome (PRRS) is a highly contagious immunosuppressive disease caused by the porcine reproductive and respiratory syndrome virus (PRRSV) that is characterized by a highly variable gene sequence and a high rate of recombination, thereby contributing to difficulties in the clinical prevention and control of this virus. Glycosylated protein 3 (GP3) is the most glycosylated protein in PRRSV, and is closely associated with the composition of PRRSV virus particles, infection, and immune evasion. This review summarizes the structural features, genetic evolutionary patterns, glycosylation of GP3 and its interactions with other PRRSV and host proteins, associations with PRRSV infection and virulence, and immunomodulatory roles. Additionally, it provides an overview of research progress on monoclonal antibodies and vaccines targeting GP3. This study aims to provide a theoretical foundation for better understanding the structure and function of GP3, of the mechanisms of PRRSV infection, and the development of novel vaccines.

## Simple Summary

GP3 protein is a crucial structural protein of PRRSV, which is highly glycosylated and plays a pivotal role in PRRSV infection, assembly, mutation, and the protective response. In this study, we present a comprehensive summary of the structural, genetic evolutionary, and protein structural and functional characteristics of GP3, along with an overview of the advancements in vaccine research. This is intended to serve as a theoretical foundation for future investigations into PRRSV GP3.

## 1. Introduction

Porcine reproductive and respiratory syndrome (PRRS), commonly referred to as “blue ear disease”, is an acute highly contagious immunosuppressive disease caused by the porcine reproductive and respiratory syndrome virus (PRRSV) that can infect pigs of different ages, although it is mainly detected in pregnant sows and piglets. Infected sows typically suffer from reproductive disorders with clinical manifestations of weak, stillborn, mummified fetuses and abortions, whereas infected weaned piglets tend to be characterized by respiratory disorder [1,2,3]. PRRS was first discovered in 1987 in the United States, at which time it was referred to as a “mysterious disease”, and subsequently spread rapidly worldwide [4]. In 1991, the Dutch scientist Wensvoot isolated PRRSV for the first time, establishing it to be an RNA virus, and named the isolated strain the Lelystad virus (LV) [5]. Subsequently, on the basis of a retrospective study of the virus, Carman et al. [6] detected PRRSV antibody positivity in samples taken prior to 1979, thereby providing evidence that PRRSV had begun to spread earlier than 1979. PRRS was first reported in China in 1995, and PRRSV was first isolated from a domestic swine population in 1996 and designated as CH-1a [7]. PRRSV has undergone mutations during transmission, leading to the evolution of HP-PRRSV, NADC30-like PRRSV, and NADC34-like PRRSV strains [8,9,10,11,12]. Globally, two major outbreaks of PRRS have documented; the first occurred in Central Europe and North America in the late 1980s [13,14], and the second in 2006, primarily affecting Asian countries such as China and Vietnam [15,16]. PRRSV exhibits a high recombination rate and mutability [17,18], making it challenging to prevent and control clinically, thereby contributing to substantial economic losses in the global pig industry, and is one of the most important infectious diseases threatening the global livestock and poultry industry, alongside other high-risk animal infectious diseases [19,20,21].

**Figure 1:**
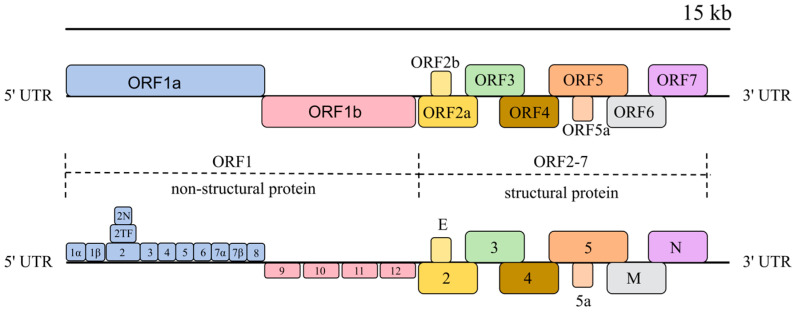
The structure of the PRRSV genome. The upper icons represent the different gene segments in PRRSV and the lower icons represent the corresponding proteins encoded by the different gene pairs in the upper layer.

PRRSV is a single-stranded, positive-stranded RNA virus within the order Nidovirales that has a capsular membrane and exclusively infects cells of the porcine monocyte-macrophage lineage [22,23,24]. The PRRSV genome is approximately 15 kb in length and, similar to other viruses in the genus Arteritis, is non-segmented. It begins with a non-coding region (5′UTR) with cap structure (5′-Cap) at the 5′ end and terminates with a non-coding region (3′UTR) and a polyadenylated tail structure (3′-polyA) at the 3′ end [25,26]. The genome consists of at least 11 open reading frames (ORFs) encoding both structural and non-structural proteins. The structural proteins (glycoprotein [GP]) are encoded by ORF2a, ORF2b, ORFs3-7, and ORF5a, while the non-structural proteins (NSPs) are encoded by ORF1a and ORF1b, along with overlapping regions such as NSP2TF and NSP2N, which overlap with the coding region of ORF1a non-structural protein 2 (NSP2). ORF1a and ORF1b are located at the 5′ end of the genome and occupy approximately 75% of the entire PRRSV genome, encoding at least 16 non-structural proteins, including NSP1α, NSP1β, NSP2, NSP2TF, NSP2N, NSP3-6 NSP7α, NSP7β, and NSP8-12. These non-structural proteins perform essential functions such as protease activity, replication, and host gene regulation [27,28,29]. Structural proteins encoded by ORF2a, ORF2b, ORFs3-7 and ORF5a at the 3′ end include GP2, E, GP3, GP4, GP5, GP5a, the viral matrix protein M, and the nucleocapsid protein N [30,31]. Among these, GP5, matrix protein M, and core coat protein N are the major structural proteins of PRRSV, whereas GP2a, GP2b(E), GP3, GP4 and GP5a are minor structural proteins. Although the minor structural proteins constitute a smaller proportion of the viral structure, they play a critical role in viral packaging and receptor recognition during host invasion [32,33] (Figure 1).

PRRSV can be categorized into two genotypes based on their genomic characteristics: type 1 (European, represented by the LV strain) and type 2 (American, represented by the VR-2332 strain). These two genotypes exhibit approximately 50% nucleotide sequence difference, with type 2 strains showing higher variability compared to type 1 [2,34,35]. According to a new bioclassification, Kuhn et al. [22] categorized PRRSV into *Suid 1 rodartevirus* (PRRSV-1) and *Suid 2 rodartevirus* (PRRSV-2). However, the International Committee for the Classification of Viruses (ICVC) have categorized PRRSV into *Betaarterivirus suid 1* (formerly PRRSV-1) and *Betaarterivirus suid 2* (formerly PRRSV-2) under Betaarterivirus, and strains of both genotypes can be further divided into several lineages [36].

**Figure 2:**
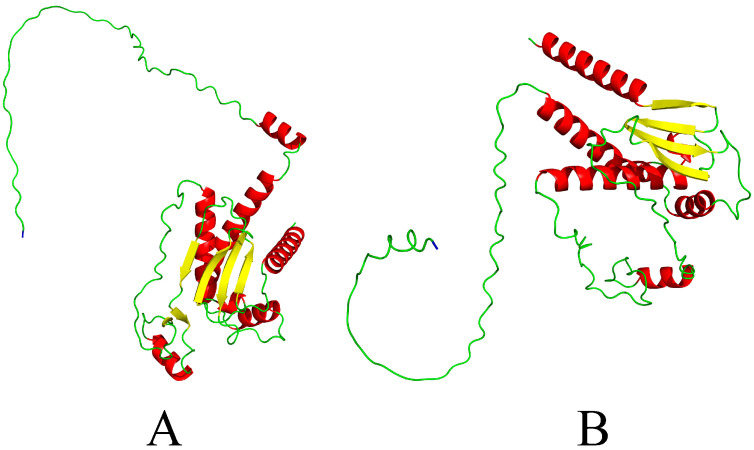
Tertiary structures of the GP3 protein predicted using AlphaFold 3: (**A**) the PRRSV-1 representative strain Lelystad Virus; (**B**) the PRRSV-2 representative strain VR2332. Red color corresponds to α-helical structure, yellow color corresponds to β-folded structure, green color corresponds to irregularly coiled structure and blue color corresponds to C-terminal end in proteins.

Mutation and recombination of genes are important for the evolution of PRRSV lineages, with new sub-lineages constantly emerging [37,38,39]. Glycosylated protein 3 (GP3) is a structural protein encoded by the ORF3 gene, consisting of a cleaved signal peptide, a highly glycosylated structural domain, a short hydrophobic region, and an unglycosylated highly variable C-terminal structural domain. GP3 is closely associated with PRRSV-related pathogenicity, replication, assembly, mutation, and the protective response to this virus [40,41] (Figure 2). The theoretical molecular weight of GP3 is approximately 27–29 kDa, although the high degree of glycosylation of the GP3 protein results in a true molecular weight of 42–50 kDa [42,43]. GP3 is a weak membrane-associated protein that tends to be characterized by differences in different virulent strains, serving as a structural protein in the LV, FL12, and Canadian QuebecIAF-Klop strains [43]. In PRRSV-2 strains, GP3 was initially believed to be a soluble secreted protein rather than a structural protein, and in 2009, it was subsequently confirmed to function as a vesicle membrane protein in both PRRSV-1 and PRRSV-2 strains, and established to be present on the surface of PRRSV-2 strains [32]. The GP3 protein can also exist as a minor GP3 fragment in the form of secreted GP3 (sGP3) that can be translocated via release from the slippery endoplasmic reticulum of PRRSV-infected cells. It has been suggested that sGP3 may function as a decoy that diverts antibodies from attacking the virus [44]. GP3 has also been identified as one of the most differentiated proteins among PRRSV strains, with nucleotide sequence differences of approximately 40%, and approximately 30% differences between different strains within the same genotype [45]. The GP3 proteins of PRRSV-1 and PRRSV-2 strains contain 265 and 254 amino acid residues, respectively, with amino acid mutations occurring predominantly in the N-terminal region, and among 35 amino acids in this region; there is only 29% identity between sequences in the two PRRSV types [24,43,46]. It is speculated that mutations in this region may contribute to differences in the virulence among strains [47,48]. The C terminus of GP3 is similarly a highly variable region that may contain antigenic epitopes that are virus-neutralized with two types of specificity [49]. GP3 has a highly variable amino acid sequence and after the GP5 protein is the most mutable structural protein in PRRSV, with a homology of approximately 54% to 60% between North American- and European-type strains [50]. The protein also has a high degree of glycosylation and tends to be poorly conserved, which has important implications with respect to PRRSV infection and assembly [51,52]. It has also been established that the GP3 protein in American strains contains two additional conserved antigenic functional regions, the variations in which may occur as a consequence of selective mutations in PRRSV, which may in turn alter the antigenicity of GP3 and ultimately affect its response to positive sera [53].

## 2. Analysis of the Genetic Evolution of GP3 Protein

To facilitate an analysis of the genetic evolution of the GP3 protein, we selected the nucleotide sequences of the GP3 of 50 PRRSV strains from the GenBank database on the NCBI website (https://www.ncbi.nlm.nih.gov, accessed on 25 October 2024), all of which were classical, epidemic, or vaccine strains of different genotypes isolated in different regions from 1991 to 2024 (Table 1).

Analysis of the 50 GP3 nucleotide sequences for nucleotide homology (Table 2, Supplementary Figure S1) revealed that the PRRSV-1 strains show 80.0% to 99.6% sequence homology. Among these strains, the IT14-32_P85 and WestSib13 strains were found to have lowest nucleotide homology at 80.0%, whereas the MLV-DV-Netherlands and BH1 strains have the highest homology of 99.6%. Comparatively, PRRSV-2 strains have homologies ranging from 81.2% to 100%, among which the GD20220302 and Ingelvac ATP strains have the lowest homology, whereas the TZJ226 and TZJ3005, MLV RespPRRS Repro and BJ-4, and SP and Prime Pac strains each show 100% homology. With respect to GP3 nucleotide sequence homologies between the PRRSV-1 and PRRSV-2 strains, we recorded values of between 61.75% and 68.2%, with strains WK730 and AUT22-97 showing the lowest value, and the HeN-L1 and KZ2018 strains having the highest homology.

The amino acid sequences of GP3 in the 50 strains were similarly compared for amino acid homology (Table 2, Supplementary Figure S2). Sequences among the PRRSV-1 strains were found to be 73.6–99.2% homologous, with the highest homology between the MLV-DV and BH1 strains, and the lowest between the IT14-32_P85 and JBNU-19-E01 strains. Comparatively, we detected homologies of between 74.1% and 100% for PRRSV-2 strains, among which strains R98 and WK730 showed the lowest homology, with the MLV RespPRRS Repro and BJ-4, SP and Prime Pac, and TZJ226 and TZJ226 strains all being 100% homologous. In terms of the two genotypes, GP3 amino acid homology between PRRSV-1 and PRRSV-2 strains ranged from 51.6% to 63.8%, with the lowest homology of 51.6% between the JBNU-19-E01 (PRRSV-1) and R98 (PRRSV-2) strains, and the highest homology of 63.8% between the WestSib13 strain (PRRSV-1) and ATCC VR-2332 (PRRSV-2), HeN-L1 (PRRSV-2), SP (PRRSV-2), and Prime Pac (PRRSV-2) strain.

**Figure 3:**
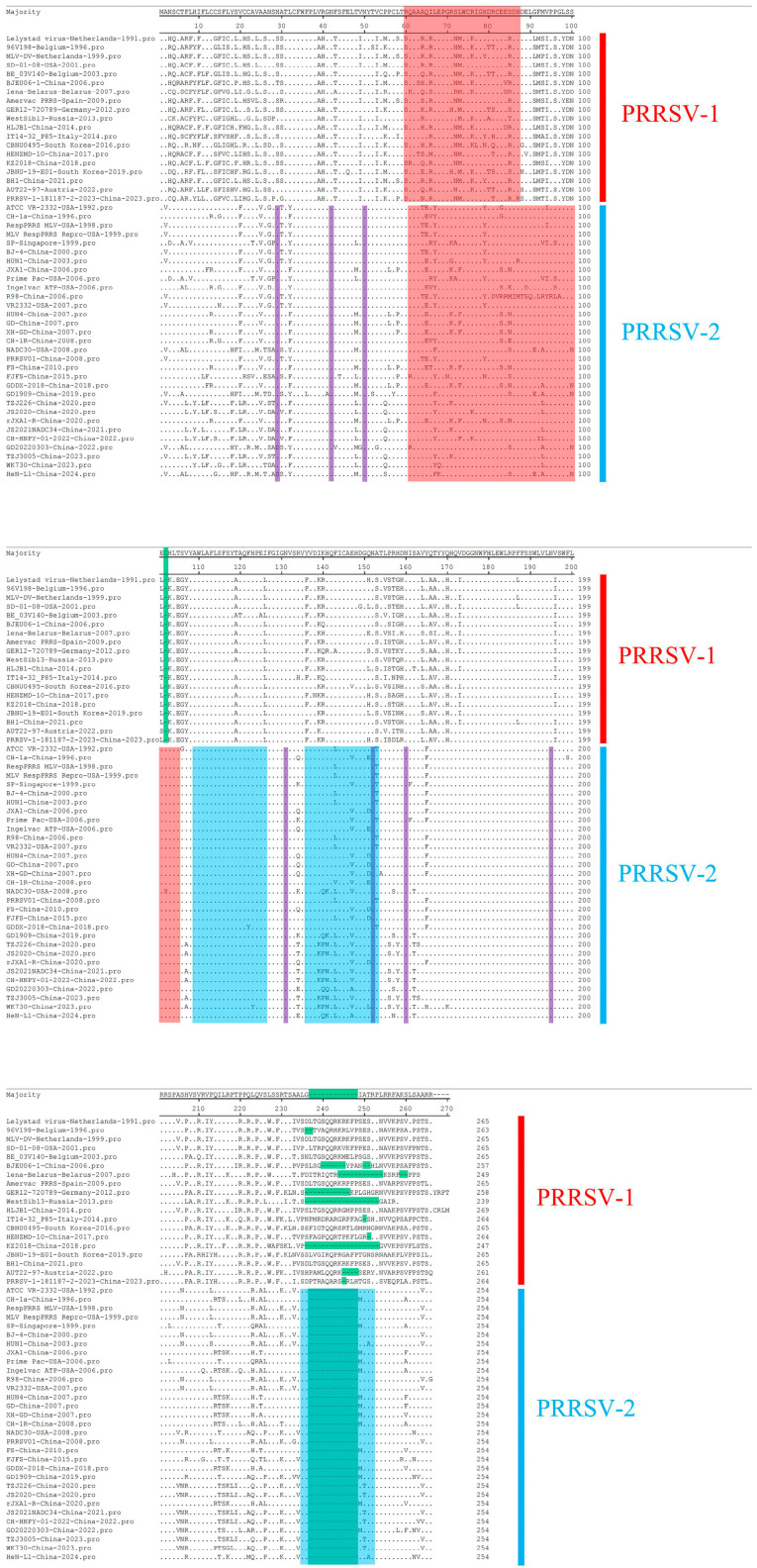
Comparison of the amino acid sequences of the selected 50 strains. Red indicates B-cell epitope regions, blue indicates T-cell epitope regions, green indicates amino acid deletions, and purple indicates glycosylation sites. Amino acid sequence alignment was performed using the sequence comparison and editing functions of the MegAlign function within DNAStar software (version 7.0, Madison, WI, USA).

Comparisons among the GP3 amino acid sequences of the 50 strains (Figure 3) revealed 265 amino acids and seven potential glycosylation sites (N27, N42, N50, N130, N151, N159, and N194) in the PRRSV-1 type strains, and 254 amino acids and seven potential glycosylation sites (N29, N42, N50, N131, N152, N160, and N195) in the PRRSV-2 type strains [24,43,54]. The differing degrees of mutation in the PRRSV-1 strains have given rise to changes in the number of amino acids and the positions of glycosylation sites in the 19 strains for which sequence analysis was performed, with the actual number of amino acids in these 19 strains ranging from 247 to 269, among which the WestSib13 and the HLJB1 strains contain the fewest and most amino acids, respectively. All PRRSV-1 strains had a single-site deletion at amino acid position 102, and 10 strains, including 96V198, had multiple site deletions at positions 236 to 265. Additionally, the degree of variation at positions 1 to 30 and 230 to 269 was found to be significantly greater than in other regions, thereby indicating a more pronounced variation in the amino acid head and tail ends of PRRSV-1 strains compared with the central region. All PRRSV-2-type strains for which amino acid sequence analysis was performed contained 254 amino acids. However, we detected no amino acid additions among these strains, no deletion at position 102, and 12 amino acids were missing from positions 236–248 compared with the classical PRRSV-1 LV strain, which is consistent with the findings of Gonin et al. [43,47]. As a consequence, the GP3 proteins of the European-type PRRSV strains are characterized by a hydrophilic C terminus, whereas the GP3 proteins of the American-type strains are amphiphilic proteins that have both hydrophilic and hydrophobic properties. Among the assessed PRRSV-2 strains, GD20220303 and HeN1-L1 were identified as the only strains with the N29→D29 and N29→S29 mutations, respectively, and none of the remaining strains had glycosylation site mutations. Additionally, we found that among the PRRSV-2 strains, amino acids 1–127 showed a higher degree of mutation than those at other loci. Comparatively, PRRSV-1 strains showed a higher degree of mutation than PRRSV-2 strains, and have an additional 12 amino acids at position 236 to 248, along with a single amino acid deletion at position 102, which may account for differences in the virulence of strains of the two PRRSV genotypes. Furthermore, compared with the PRRSV-1 strains, we found PRRSV-2 strains to be more uniformly mutated. Among PRRSV proteins, GP3 was identified as being the most highly glycosylated, and also tends to show a lesser degree of conservation among the conserved PRRSV protein [55,56,57]. Of the other six more conserved glycosylated proteins of the PRRSV-1 and PRRSV-2 strains, only the first glycosylation site is mutated, with the N27 site in PRRSV-1-type strains being slightly more mutated than the N29 site in PRRSV-2-type strains.

**Figure 4:**
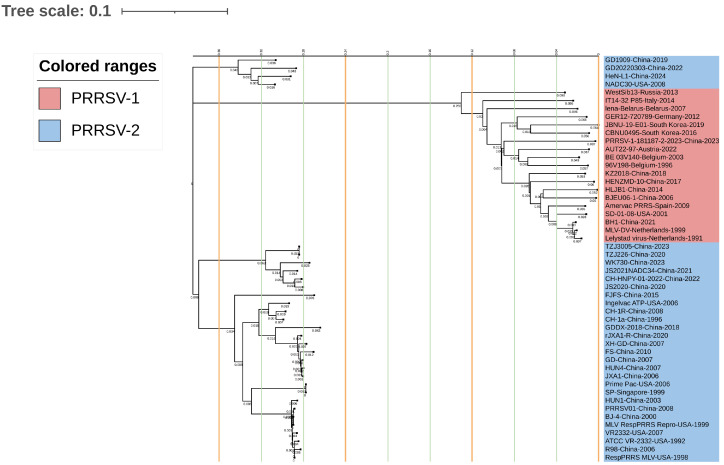
A phylogenetic tree of the GP3 gene constructed based on Maximum Likelihood method using the MegAlign function in DNAStar software (version 7.0, Madison, WI, USA), with alignment of sequences performed using Clustal W method with default parameters in MEGA (version 7.0) for 1000 iterations. PRRSV-1 and PRRSV-2 strains are labeled in red and blue, respectively.

The nucleotide sequences of GP3 in the 50 assessed strains were used to construct a phylogenetic tree based on the Maximum Likelihood (ML) method (Figure 4). Predictably, the results indicated that the PRRSV strains can be divided into two major branches, PRRSV-1 and PRRSV-2. Among the former strains, WestSib13 was found to be the most genetically distant from the MLV-DV strain, whereas the LV strain was closest and was located on the same branch as the BH1 and MLV-DV strains. Among the PRRSV-2 strains, GD1909 was closest to the RH1 and MLV-DV strains, mapping on the same branch. Conversely, the GD1909 strain was observed to be the most distantly genetically related to the RespPRRS MLV strain. A further PRRSV-2-type strain, HeN-L1, which was recently isolated in 2024, was found to be genetically closest to the NADC30 strain, whereas CH-1a, the earliest Chinese isolate, was identified as being genetically closest to the CH-1R strain, which was located on the same branch as the Ingelvac ATP strain.

The genotypic classification of PRRSV strains may vary depending on the computational methods used to analyze different gene sequences. For example, the TZJ226 strain is classified as PRRSV-2 in the phylogenetic tree based on N protein, while PRRSV-1 in the phylogenetic tree is based on the GP3 protein [58]. PRRSV-2 strains in China comprise lineages 1, 3, 5, and 8, which differ in terms of epidemic time, region, and extent [59,60]. Comparatively, whereas PRRSV-1 strains can be categorized into four lineages [61], PRRSV-2 strains have been classified into eleven lineages [62], with lineages of the strains of both genotypes being further subdivided into larger numbers of sub-lineages [63]. On the basis of an analysis of N-glycosylation of the GP5 protein, Zhang et al. [60] differentiated the spectrum of PRRSV, and found that fluctuations in the glycosylation pattern of this protein coincided with the epidemiological trends of PRRSV in China, thereby providing a novel perspective with respect to spectrum delineation of the PRRSV type-2 strains.

## 3. Glycosylation

GP3 is the most glycosylated and among the most antigenic of the PRRSV proteins [56], and its glycosylation functions similarly to that of the GP5 protein in terms of immune evasion [64,65]. In both genotypes of PRRSV, the GP3 protein has seven potential glycosylation sites [42], N27, N42, N50, N130, N151, N159, and N194 in PRRSV-1-type GP3 proteins, and N29, N42, N50, N131, N152, N160, and N195 in tPRRSV-2-type GP3 proteins, of which the first six amino acid sites have been confirmed to undergo glycosylation, whereas the N195 site, which may lie in the GP3 transmembrane region, is considered unlikely to undergo glycosylation [52]. Glycosylation of sites N42, N50, and N131 has been suggested to be associated with the formation of viral particles and in PRRSV-2 strains is required for the production of infectious particles [52,66]. Mutations in the remaining glycosylation sites have also been indicated to influence viral infectivity, although they have no significant effects on virus production or proliferation [67]. Additionally, N131 has been associated with the immune escape process and can stimulate the production of high levels of neutralizing antibodies in host organisms [33].

Site-directed mutagenesis of different GP3 glycosylation sites performed to rescue the virus have been found to have differing effects [52]. In this regard, different glycan structures, modification types, and linkages can confer differences in the capacity of proteins to recognize different receptors, and alterations to one glycosylation site may modify not only the conformation of that site but also have an influence on the glycan chains of other glycosylation sites in the same viral proteins. The ensuing cascade of conformational changes may in turn inhibit the ability of membrane proteins to recognize receptors, or even alter the type of receptor recognition, thereby affecting viral infectivity [68]. Furthermore, whereas mutation of PRRSV GP3 at a single glycosylation site can rescue a mutated virus, strains with simultaneous mutations at multiple glycosylation sites may not be successfully rescued [69]. In this regard, Liu et al. [70] found that targeting single sites (N29, N42, or N131) of GP3 in mutant strains of PRRSV can rescue viruses; however, they were unable to rescue strains with simultaneous mutations at multiple sites (N29+N42, N29+N131, and N29+N50+N131), which is consistent with the findings of Das et al. [52], who found that recombinant viruses with mutations in N29, N42, and N131 could not be rescued, indicating that these three glycosylation sites are important for the viability of virus particles. By sequencing such unsalvageable viruses, Xu et al. [71] found that the sequences of these viruses differed from those of the parental strains only with respect to the mutated glycosylation site, thereby indicating that the mutation or deletion of a single site in the ORF3 gene may cause structural changes and modify the configuration of other sites due to a spatial site-blocking effect, which would contribute to altering the glycosylation pattern of the entire GP3 protein, thus promoting changes in the function and activity of the GP3 protein. It is also conceivable that the mutation or deletion of a single site may have only a weak effect on the overall glycosylation of the GP3 protein, given the potential compensatory effects of other glycosylation sites in alleviating the effect of single-site mutations. Collectively, these findings would thus tend to indicate that whereas changes in a single glycosylation site would probably have limited effects on the GP3 protein as a whole, the simultaneous mutation of multiple glycosylation sites would have a more substantial impact on the biological activity, spatial folding, and conformation of the GP3 protein. This in turn would interfere with the host receptor recognition capacity of PRRSV, thereby preventing fusion between the vesicle membrane of PRRSV and the host cell membrane, ultimately resulting in a loss of infectivity.

Notably, it also been established that certain freshly isolated strains typically lack deletions or mutations of glycosylation sites within the ORF3 gene; deletion of glycosylation sites would occur following several generations of cell passaging. Nevertheless, after infecting pigs with strains carrying such deletions, it was found that deleted glycosylation sites could be recovered, which may indicate the occurrence of a strong selective pressure to retain certain glycosylation sites, which may play essential roles immune evasion due to stimulation of immune-related molecules produced by the host immune system [33]. Alternatively, this may be associated with the fact that the virus needs to adopt a different mode of infection or receptor recognition in response to differences in the receptors of the infected cells, which may necessitate glycosylation-related modification to facilitate the infection of different cell types [72].

## 4. Interactions Between GP3 and Other PRRSV Proteins

Among the other PRRSV proteins, GP5 and M are major envelope proteins, glycosylation of the former of which plays roles in PRRSV escape similar to those in GP3, and also contribute to blocking or minimizing viral-neutralizing antibody responses [65,73,74]. GP2, GP2b, GP3, GP4, and GP5a are considered minor envelope proteins involved in PRRSV infection and neutralizing antibody production [75], whereas GP2a serves as a linkage mediator that can link GP3, GP4, GP5 and M proteins to form protein aggregates. In both PRRSV genotypes, GP2a, GP3, and GP4 also act as trimers in formation of the PRRSV vesicle membrane and are involved in PRRSV infection and neutralizing antibody production [52,76]. GP2, GP3 and GP4 together with non-glycosylated E proteins can form heterotetramers of heterologous proteins in cells that have been infected with PRRSV, which, although non-essential for the formation of PRRSV virus particles, play pivotal roles in the infection process. Moreover, it has been established that GP3, GP4 and GP5 can undergo strong interaction and form a GP2a-GP3-GP4-GP5 multimeric protein complex, in which GP4 plays a key role in mediating binding [49].

## 5. Interactions Between GP3 and Host Proteins

**Figure 5:**
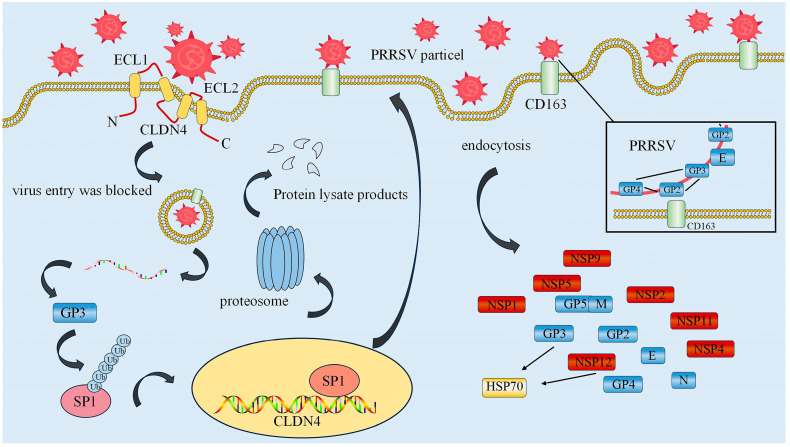
Interactions between the GP3 protein and other proteins. The protein interaction diagrams were generated using ScienceSlides software (version 2016).

Different PRRSV proteins can interact with host cell proteins via different mechanisms, and among the main roles of PRRSV glycoproteins is that of endogenous and exogenous receptors that mediate processes such as viral invasion and release (Figure 5) [74]. Certain envelope glycoproteins of PRRSV, which are often antigenic, can selectively recognize and bind to host cell receptors, thereby promoting fusion between viral vesicle membranes and host cell membranes, thus initiating host cell invasion [77]. In this context, it has been established that the small extracellular loop (ECL2) of host cell cloned protein 4 (CLDN4) can bind to viral GP3, thereby restricting the entry of extracellular PRRSV particles into host cells, and effectively blocking the absorption and invasion of PRRSV, whilst neutralizing the virus. However, Ding et al. [78] have proposed that GP3 promotes PRRSV infection by mediating the transcriptional regulation of CLDN4, whereby GP3 promotes the ubiquitinated degradation of transcription factor SP1, thereby downregulating the transcription of CLDN4. Furthermore, the lipid stroma of the host cell membrane has been shown to be associated with the replication and release of PRRSV, and disruption of the cytosolic stroma inhibits the entry of PRRSV into cells [79]. Yang et al. [80] found that the entry of PRRSV into the host cell is dependent on the lipid stroma, and that PRRSV GP3 and GP4 play key roles in this process.

CD163, encoded by the CD163 gene, on the surface of PRRSV-susceptible porcine alveolar macrophages (PAMs) has been identified as an essential host cell receptor protein associated with PRRSV host cell invasion in conjunction with porcine sialic acid adhesin receptors. It is believed to function via a low pH-dependent lattice protein-mediated endocytosis pathway in response to co-activation of certain cofactors, and mainly acts during the stages of PRRSV viral particle decapsidation and release of the viral genome [81,82]. Notably, pigs lacking the CD163 gene are resistant to PRRSV infection [83,84,85,86]. In conjunction with the secondary envelope proteins GP2 and GP4, GP3 is one of the major determinants of the high or low cellular affinity of PRRSV. These three proteins form a heterodimer of heterologous proteins, linked via disulfide bonds, that interacts with the CD163 protein to facilitate host cell entry, and in the absence of this heterologous protein trimer, the virus is unable recognize and interact with host cell receptors, indicating that the GP2-GP3-GP4 protein complex plays an essential role in host cell infection [87,88,89]. Additionally, GP4 has been established to function as a fusion mediator involved in the process whereby the protein aggregates formed by GP2a, GP3, GP4, GP5, and M interact with CD163 receptors and fuse with susceptible host cell membranes, ultimately mediating PRRSV infection [90]. The antigenicity of GP3 is mainly in the induction of cellular immunity, and it can effectively stimulate the host to produce effective antibodies, but it is not as effective as GP5 [91,92].

Heat shock proteins (HSPs) are potent immune adjuvants of the adaptive immune system that contribute to enhancing innate immunity [54,93,94], and are also involved in the regulation of apoptosis and antiviral immune responses [95,96]. Among these proteins, HPS70, a highly conserved protein with high interspecies homology, has an efficacy comparable to that of helper and protective antigens. As an anti-apoptotic protein, elevated levels of HPS70 can contribute to complete inhibition of the endogenous apoptotic pathway, which protects against cytotoxicity induced by a range of factors, and may be utilized by PRRSV to ensure that viral replication is not detected, whilst inhibiting apoptosis [97,98]. GP3 can participate in interferon-induced downstream signaling processes, and the interaction between HSP70 and GP3 of HP-PRRSV endoviral strains has been found to induce the production of IFN-γ and IL-4 in porcine serum, thereby enhancing the immune response, which can effectively protect pigs from PRRSV infection, a process that may provide a basis for the construction of recombinant adenovirus vaccines [99,100].

## 6. GP3 with Its Distribution and Virulence Variation in PRRSV Infection

Immunofluorescence analysis has revealed that GP3 is distributed among different sites within cells during PRRSV infection. Whereas small amounts of GP3-specific fluorescence have been detected on the cell membrane at 24 h after PRRSV infection of susceptible cells, a majority of the fluorescence appeared on the surface of internal membrane, with no specific fluorescence being detected in the interstitial fluid or cytoplasm, indicating that although mainly present within the cell at this stage, it is probably confined to the endoplasmic reticulum [101,102]. The extensive fluorescence observed in the cell membrane at 36 h after infection would tend to indicate that a proportion of the virus is released from the cell during the process of PRRSV proliferation, and after 48 h, more intense fluorescence has been detected externally to the cell membrane, indicating that a proportion of the GP3 is transferred out of the cell with the release of PRRSV and occurs in a free state, thereby providing evidence to indicate this protein may be involved in PRRSV assembly and virus particle release [103].

GP3 is highly mutated protein, and compared with classical strains, the GP3 of HP-PRRSV strains is characterized by eight mutated amino acid sites, among which one is located at the end of the C terminus, four are non-conservative mutations, and a conserved mutation at amino acid 83 may be associated with the attenuated virulence of PRRSV vaccine strains [104,105]. In this regard, Wu et al. [106] have shown that although an attenuation of Chinese CH-1a strain transmission is not associated with alterations of amino acids at position 13 and 151, the mutations at amino acids 38 and 146 may have a pronounced influence on the virulence of this virus. Virulence-associated mutations in PRRSV may also have a para-cumulative effect, thereby making it difficult to differentiate between local isolates and weakly virulent vaccine strains originating from strain VR-2332 using RFLP methods alone [107]. Additionally, mutated GP3 glycosylation sites have been established to influence virulence, among which N29, N152, and N160 do not influence the capacity of the virus to neutralize antibodies, whereas deletion of N131 in GP3 and GP5 has been found to protect the virus from neutralizing antibodies [33,108].

## 7. Involvement of GP3 in Host Immune Responses

The envelope glycoproteins of PRRSV are often antigenic and can selectively recognize and bind to host cell receptors, thereby promoting fusion of the viral vesicle membrane with the host cell membrane and thus facilitating subsequent host cell invasion [75]. Despite the low amounts of GP3 in PRRSV, the antigenicity of this protein is high and polymorphic [33,109]. With a view toward determining the immunogenicity and mechanisms of action of GP3, Jiang et al. [73] used an adenoviral live vector to construct rAd-GP3 and rAd-tGP3 recombinant live vector vaccines, and accordingly found that the expression of the first 64 amino acids in the structure of the GP3 protein had no affect evident effect on antigenicity. GP3 also induces the production of neutralizing antibodies [33,110,111] and generates a protective immune response specific for PRRSV, and, accordingly, is often considered to be associated with host cell body protective immunity [34,102,112]. The N-terminal glycosylation site of GP3 has been demonstrated to interfere with host neutralizing antibody production, and in HP-PRRSV strains, the GP3 protein contains at least one neutralizing epitope that may be involved in PRRSV re-infection [33,113], and similarly, GP2, GP3, and GP4 can interact with the cellular receptor CD163 to induce the production of neutralizing antibodies [114].

There are six theoretical prediction schemes for B cell antigen epitopes, namely the hydrophilicity, accessibility, antigenicity, plasticity, charge distribution profile, and secondary structure prediction schemes, although the accuracy of these schemes is not high [115]. The M, GP2a, GP3, GP4, and GP5 proteins of PRRSV all contain viral neutralization epitopes, among which a region of GP3 has been found to functionally interact with host cells, thereby indicating that antibodies directed against this region could contribute to viral neutralization [34,113]. Tandem expression of GP3 and GP5 has been shown to be effective in stimulating the production of high levels of neutralizing antibodies against PRRSV in immunized animals, and also enhances the GP-mediated stimulation of the host immune response [116]. Although the GP3 proteins of both PRRSV-1 and PRRSV-2 strains contain linear antigenic regions, antibodies directed against a majority of the epitopes are unable to effectively neutralize the virus [53,117]. However, B-cell epitopes of the region spanning amino acids 60 to 87 in the GP3 proteins of both PRRSV-1 and PRRSV-2 strains can induce neutralizing antibodies. Additionally, the PRRSV-2-type GP3 protein has four overlapping (61QAAAEVYEPGRSLWC75, 71RSLWCRIGHDRCSED85, 81RCSEDDHDDLGFMVP95 and 91GFMVPPGLSSEGHLT105) and strong immunoreactive B-cell epitopes in the region spanning amino acids 61 to 105 [32,42,118,119,120]. In further studies, using phage techniques, Tao [121] identified antigenic epitopes located at amino acid positions 64 to 70 in the GP3 protein of the SC-GY strain and provided evidence to indicate that L65S67T68 may play an important role in GP3 linear epitopes. Additionally, Zhou et al. [53] identified 58–72, 73–87, 88–101, 102–115, 50–65, 66–81, 80–95 and 94–109 of GP3, above eight groups of antigen recognition sites.

Although there have been a number of studies that have examined the B-cell epitopes of GP3, comparatively few have focused on GP3 T-cell epitopes. Among those studies that have been conducted, Che [122] initially localized a T-cell epitope within a region between amino acid positions 120 and 209 in a truncated GP3 protein of the BJ-4 strain. GP3 can stimulate variable weak cellular immunity, although high humoral immunity, and, consequently, ORF3 is considered a promising target gene for the development of a new generation of highly efficient genetically engineered vaccines [123]. GP3-induced T-cell immune responses have been established to have immune protective effects, and it has been found that the M, N, GP2a, GP3, GP4, and GP5 proteins can stimulate T-lymphocyte proliferation [124]. Furthermore, on the basis of a screening of vaccine strains, Diaz et al. [125] found that protective vaccine strains can stimulate the production of a strong cellular immune response, mediated via the secretion of high levels of IFN-γ. Consequently, the levels of PRRSV-induced immune protection may be determined by the cellular immunity induced by this virus.

## 8. Application of GP3 in Detection of PRRSV

Given the diverse signs of infection, diagnosis of PRRSV based on clinical symptoms alone is typically difficult; however, pathogenetic and serological tests have been established as effective diagnostic approaches [126,127]. Pathogenetic methods mainly consist of virus isolation and characterization, which is the most classical method for the identification and diagnosis of PRRSV. PRRSV strains can be isolated using a variety of cell lines such as PAM, CL 2621, MA104, MARC-145, and ZMAC, but the isolation success rates of type 1 and type 2 PRRSV in different cell lines are not consistent, and VR-2332 (PRRSV-2) can be grown in MA104 cells, but not in PAM [128,129]. In addition, ZMAC cells also have different isolation success rates for different spectral strains of type 2 PRRSV, so a variety of cell lines should be selected when isolating PRRS [130]. Serological methods with high sensitivity, good specificity and simple operation are the most widely used methods for laboratory detection of PRRSV, whereas indirect ELISA assays are widely used for rapid clinical detection, such as the indirect ELISA method for PRRSV antibodies established using GP3 and GP4 protein peptides as the encapsulated antigens.

Different monoclonal antibodies can be prepared against strains with different PRRSV genes, and those raised against the GP3, GP4, and N proteins are characterized by differential reaction with European- and American-type strains, thereby indicating differences in the antigenic signatures of these different groups of strains, and also between different strains within the same type [18,47]. The majority of currently used monoclonal antibodies raised against PRRSV target structural proteins and are widely used to detect PRRS, whereas antibodies against N proteins are the most abundant among the monoclonal antibodies obtained using mice immunized with PRRSV. Comparatively, relatively few such antibodies are directed against the M, GP2, GP3, and GP4 proteins. Studies that have assessed the effects of monoclonal antibodies with neutralizing effects, including those directed against the GP3 protein of LV strains, have established that these agents can contribute to the elimination of free extracellular viral infections, whereas specific antibodies directed against ORF3 have been found to react with only a fraction of the assessed virulent strains [41,131,132]. Additionally, phage display techniques have revealed that peptides synthesized from a mimic antigenic epitope of the PRRSV GP3 protein are effective in inhibiting PRRSV and enhancing the binding of monoclonal antibodies. Furthermore, using monoclonal antibody technology, Cancel-Tirad et al. [113] established that the GP3 protein contains neutralizing antibody epitopes, which can induce host production of neutralizing antibodies, and that monoclonal antibodies directed against GP3 can inhibit the replication of PRRSV in alveolar macrophages. Similarly, Meulenberg et al. [133] found that the application of a monoclonal antibody against GP3 had a protective effect against PRRSV infection, whereas using a monoclonal antibody against the GP3 protein of a UK isolate, Drew et al. [134], who analyzed antigenic fragments of the conserved and variable regions of ORF3, found that the C-terminal end of the ORF can act as a highly variable region, and is susceptible to immunization. Additionally, Liu et al. [92], who obtained an antigen by truncating the GP3 protein, which involved the deletion of fragments of the hydrophobic regions at the N and C termini of the protein, found that although it overcame the drawbacks of using the poorly expressed full-length ORF3 gene, the monoclonal antibody thus obtained showed only a weak interaction with virus-infected cells. Moreover, Sun [135] used unpurified and vital virus-immunized mice to prepare monoclonal antibodies against HP-PRRSV GP3 and thereby identify antigenic epitopes. The 4G5 monoclonal antibody thus obtained was shown to recognize an epitope in the aa74WCRIGHDRCS83 region, and there may be multiple epitopes in this region, which is two amino acids less and one amino acid variant (S83→G83) at the C-terminal compared with the aa74WCRIGHDRCGED85 region identified by Zhou et al. [51,53].

## 9. The Role of GP3 in Vaccine Preparation

Given the different extents of antigenic variation between the different PRRSV genotypes and among strains within the same genotype, the clinical use of a single vaccine strain would be ineffective in providing immunity against PRRSV infections and the prevention of epidemics [18,47,136]. Consequently, different regions in the prevention of PRRS should be used at the most recent time of the epidemic as well as local strains and antigenically similar strains of vaccine preparation. Given that the glycosylated virus proteins interact with host cell receptors to initiate infection and mediate viral entry into host cells, glycoproteins have traditionally been a key focus in vaccine research. Moreover, glycoproteins have receptor binding sites and antigenic epitopes that play important roles in inducing host immune responses and immune evasion processes [137]. The subunit vaccines based on GP3 and GP5 have been established to have good immunogenicity and can induce the production of neutralizing antibodies. Notably, GP3 is associated with the induction of cellular immunity, thereby identifying this protein as a good candidate for the development of recombinant subunit vaccines [138]. Although the value GP3 in this regard is intermediate between that of GP4 and GP5, it induces the highest levels of neutralizing antibody production during the early stages of infection, and it is only in the latter stages that the GP5 is more effective than GP3 and GP4 in this role. Moreover, given that GP3, GP4, and GP5 act synergistically in inducing neutralizing antibodies, administering a combination of vaccines based on these proteins would be considerably more effective than using the respective single-protein-based vaccines [139]. Similarly, GP3 can be used in tandem with other genes to construct vector vaccines, and whereas the neutralizing antibody titers produced using purified GP3 or GP5 protein alone are low, when GP3, GP4, and GP5 are recombined in tandem or separately into adenoviral vectors, the recombinant viruses have been demonstrated to show good immunogenicity, and the monoclonal antibodies raised against GP3 do not cause antibody-dependent enhancement. Additionally, compared with vaccines based on inactivated or attenuated viruses, it has also been established that recombinant adenoviruses expressing tandem proteins have superior immune effects [72].

Plana et al. [102,138], immunized pregnant sows with baculovirus-expressed GP3 protein, and found that the vaccine provided 68.4% protection, and could provide high protection in the presence of low levels of antibodies, and it is accordingly hypothesized that GP3 would not lead to, or only elicit, lower levels of humoral immunity, but would stimulate certain levels of cellular immunity [121]. Liang et al. [140] cloned the ORF3 gene and constructed the DNA vaccines pSCA-VPm3 and pSCA-VP6 expressing GP3 protein, the efficacies of which were evaluated by immunizing mice, and consistent with the findings reported by Plana et al. [102,138], they demonstrated that although the neutralizing antigenicity of GP3 was notably weak, it could still induce a high level of cellular immune response. Furthermore, Bai et al. [141] successfully divided a truncated GP3 protein of the HuN4 strain of HP-PRRSV into three segments for expression in *Escherichia coli* and found specific antibodies against these three segments in serum. Similarly, Xu et al. [104] heterologously expressed the GP3 protein from the HuN4 strain using *E. coli* and demonstrated that GP3 has high immunogenicity, and, using GP3 as a target antigen, Zhao et al. [123] applied recombinant technology and the balanced lethal system of mouse for *Salmonella typhimurium* to express GP3 protein, on the basis of which they constructed the first orally available live-carrier bacterial vaccine, and demonstrated the immunogenicity of the GP3 protein. In vitro characterization studies have also demonstrated that the strain (X4550[pYA3341-ORF3]) can stably carry the recombinant plasmid and can be continuously and safely transmitted in vivo and ex vivo in the absence of any pronounced side effects, whereas immunization tests using mice indicated that the recombinant vaccine strain can induce specific immunity. In further studies, Wang et al. [142] successfully constructed and expressed a fusion protein recombinant plasmid, designated pcDNA3.1-GP3-GP5-M, which was demonstrated to have good immunogenicity, and recombinant baculovirus-expressed GP3 or recombinant poxviruses expressing GP3 and GP5 have been shown to elicit partial immune responses in animals [102], whereas Zhang et al. [143] found that a recombinant heterologous protein poxvirus vaccine based on GP3-GP5 can enhance the intensity of humoral and cellular immune responses and provide effective protection. Bastos et al. [144] similarly used baculoviruses expressing only the GP3, GP5, and M proteins, and also found that the GP3 and GP5 proteins had certain protective effects when immunizing pregnant sows, and Xu et al. [71] were the first to insert genes encoding the GP3 and PCV-2 Cap proteins into a baculovirus surface display transposable vector, which was used to infect Sf-9 cells to obtain the recombinant baculovirus BacSC-Dual-ORF3-ORF2, and they accordingly found that the recombinant proteins could stimulate strong humoral and cellular immune responses.

## 10. Discussion

The GP3 protein has been established to be a highly glycosylated, although poorly conserved, PRRSV structural protein that plays important roles in PRRSV infection, assembly, mutation, and induction of immune responses. It has been identified as the second most mutable protein in PRRSV, with the amino acid sequences of the PRRSV-1 and PRRSV-2 strains being mutated to varying degrees, although the extent of mutation in PRRRSV-2 strains tends to be low and amino acid variant sites are more dispersed than that in PRRRSV-1 strains. Moreover, whereas no amino acid additions have been detected in PRRRSV-2 strains, deletions at certain sites have contributed to certain differences in the properties of the GP3 proteins in strains of the two genotypes. GP3 has also been established as the most highly glycosylated among PRRSV proteins, with the strains of both genotypes being characterized by seven relatively conserved glycosylation sites, which have been found to play roles in immune evasion similar to those of comparable sites in the GP5 protein. Moreover, mutation of single and multiple glycosylation sites has been observed to have differing effects on the efficacy of virus rescue. Recently, it has been found that certain PRRSV-2 strains have amino acid mutations at the N29 glycosylation site of GP3, which is assumed to be highly conserved. This could thus provide evidence to indicate the imminent emergence of new mutation characteristics and new mutation profiles among PRRSV-2 strains. Together with the findings that N-glycosylation of the GP5 protein can be used to distinguish PRRSV profiles, it is reasonable to infer that determining fluctuations in the patterns of GP3 glycosylation could serve as an effective approach for assessing the epidemiological trends of PRRSV in China.

In addition to interacting with host cell proteins, GP3 can also link and interact with other PRRSV proteins, forming different protein complexes with GP2a and GP4, respectively, which constitute the vesicle membrane of PRRSV and play roles in the induction of neutralizing antibodies. With respect to its interaction with host proteins, by promoting the degradation of SP1, GP3 has been established to down-regulate the transcriptional level of CLDN4, thereby contributing to the promotion of PRRSV infectivity, and the GP2-GP3-GP4 protein complex can react with CD163 to facilitate the entry of PRRSV into the host cell. Additionally, it has been found that GP3 plays a role in interferon-induced downstream signaling processes, and the interaction between GP3 and HSP70 in HP-PRRSV strains has now been established to induce a strong immune response in pigs. To date, however, there has been no conclusive evidence to indicate the involvement of GP3 in the apoptotic process. Nevertheless, given that HP-PRRSV strains remain prevalent in China, further research on HP-PRRSV GP3 could provide valuable insights for the development of new clinical drugs.

Although, the GP3 protein represents only a relatively small proportion of PRRSV particles, it is characterized by high levels of antigenicity and antigenic polymorphism, which are conducive to inducing host cell neutralizing antibodies. The findings of numerous studies have provided evidence to indicate that GP3 carries multiple linear antigenic epitopes, a majority of which are, however, unable to induce antibodies that effectively neutralize the virus. Despite being characterized as poorly conserved, an amino acid sequence comparison plot of the GP3 protein has revealed that the current amino acid sequence has a lower degree of variation with respect to the sequences of the B-cell and T-cell epitope regions compared with the N-terminus, which is the main site of amino acid mutations. Consequently, screening for efficient antigenic epitopes may contribute to the prevention and control of PRRSV. Notably, GP3 is not a protein that is traditionally used in the preparation of monoclonal antibodies against PRRSV, and is consequently rarely used in clinical testing. However, monoclonal antibodies raised against GP3 have been extensively used by scholars to study its neutralizing antibody epitopes. The neutralizing antigenicity of GP3 has been established to be very weak, primarily inducing a cellular immune response in host organisms. However, GP3 has rarely been prepared alone as a subunit vaccine for PRRSV, but rather, it is used in tandem with other PRRSV proteins to construct multiprotein vaccines, which, compared with vaccines based on single proteins, can contribute to promoting more effective immunization.

## 11. Conclusions

Despite the important roles played by the GP3 protein in PRRSV infection, to date, there have been comparatively few studies focusing specifically on this protein, with a majority of such studies examining the generative role of other PRRSV proteins when they are connected. However, given that GP3 is closely associated with the processes of PRRSV infection, immune evasion, and neutralizing antibody production, its high degree of glycosylation and multiple antigenic epitopes are deemed worthy of more comprehensive study.

## Figures and Tables

**Table 1:** Reference sequence information for the GP3 protein of 50 virulent PRRSV strains.

Year	Area	Strain	Genbank Accession Number	Genotype
1991	Netherlands	Lelystad virus	M96262	PRRSV-1
1996	Belgium	96V198	MK876228	PRRSV-1
1999	Netherlands	MLV-DV	KJ127878	PRRSV-1
2001	USA	SD-01-08	DQ489311	PRRSV-1
2003	Belgium	BE_03V140	MW053394	PRRSV-1
2006	China	BJEU06-1	GU047344	PRRSV-1
2007	Belarus	lena	JF802085	PRRSV-1
2009	Spain	Amervac PRRS	GU067771	PRRSV-1
2012	Germany	GER12-720789	OP529852	PRRSV-1
2013	Russia	WestSib13	KX668221	PRRSV-1
2014	China	HLJB1	KT224385	PRRSV-1
2014	Italy	IT14-32_P85	MK024326.1	PRRSV-1
2016	South Korea	CBNU0495	MZ287327	PRRSV-1
2017	China	HENZMD-10	KY363382	PRRSV-1
2018	China	KZ2018	MN550991	PRRSV-1
2019	South Korea	JBNU-19-E01	MW847781	PRRSV-1
2021	China	BH1	OK635576	PRRSV-1
2022	Austria	AUT22-97	OP627116	PRRSV-1
2023	China	PRRSV-1-181187-2-2023	OQ856755.1	PRRSV-1
1992	USA	ATCC VR-2332	U87392.3	PRRSV-2
1994	USA	RespPRRS MLV	AF0666183.4	PRRSV-2
1996	China	CH-1a	AY032626	PRRSV-2
1999	Singapore	SP	AF184212.1	PRRSV-2
1999	USA	MLV RespPRRS-Repro	AF159149	PRRSV-2
2000	China	BJ-4	AF331831	PRRSV-2
2003	China	HUN1	AY457635.1	PRRSV-2
2006	China	R98	DQ355796.1	PRRSV-2
2006	China	JXA1	EF112445	PRRSV-2
2006	USA	Ingelvac ATP	DQ988080.1	PRRSV-2
2006	USA	Prime Pac	DQ779791.1	PRRSV-2
2007	USA	VR2332	EF536003	PRRSV-2
2007	China	HUN4	EF635006.1	PRRSV-2
2007	China	GD	EU109503	PRRSV-2
2007	China	XH-GD	EU624117	PRRSV-2
2008	China	PRRSV01	FJ175687	PRRSV-2
2008	USA	NADC30	JN654459	PRRSV-2
2008	China	CH-1R	EU807840.1	PRRSV-2
2010	China	FS	JF796180.1	PRRSV-2
2015	China	FJFS	KP998476	PRRSV-2
2018	China	GDDX-2018	MT379661.1	PRRSV-2
2019	China	GD1909	MT165636	PRRSV-2
2020	China	TZJ226	OP566682	PRRSV-2
2020	China	rJXA1-R	MT163314	PRRSV-2
2020	China	JS2020	MZ342900	PRRSV-2
2021	China	JS2021NADC34	MZ820388	PRRSV-2
2022	China	GD20220303	OQ459668.1	PRRSV-2
2022	China	CH-HNPY-01-2022	OP716076.1	PRRSV-2
2023	China	WK730	OR826314	PRRSV-2
2023	China	TZJ3005	OR826313	PRRSV-2
2024	China	HeN-L1	PQ062578.1	PRRSV-2

**Table 2:** Analysis of the homologies of the GP3 nucleotide and amino acid sequences of 50 selected PRRSV strains using the MegAlign function within DNAStar software (version 7.0, Madison, WI, USA).

Genetype		PRRSV-1	PRRSV-2
PRRSV-1	nt	80.0–99.6	61.7–68.2
	aa	73.6–99.2	51.6–63.8
PRRSV-2	nt		81.2–100
	aa		74.1–100

## Data Availability

All datasets are available in the main manuscript. The dataset sup-porting the conclusions of this article is included within the article.

## Supplementary Materials

The following supporting information can be downloaded at: https://www.mdpi.com/article/10.3390/ani15030430/s1, Figure S1: Analysis of nucleotide homology among 50 strains of PRRSV; Figure S2: Analysis of amino acid homology among 50 strains of PRRSV.
